# Physics-informed hierarchical transformer for wearable sensor-based gait fatigue assessment

**DOI:** 10.3389/fpubh.2026.1794241

**Published:** 2026-04-13

**Authors:** Kai Ding, Zhi Li, Xiang Zou, Jingdong Jia, Chenlin Li, Zijing Jiang

**Affiliations:** 1 School of Physical Education, Xi'an University, Xi'an, China; 2 Shaanxi Provincial Joint Laboratory of Artificial Intelligence, Xi'an University, Xi'an, China; 3 School of Physical Education, Shaanxi Normal University, Xi'an, China

**Keywords:** biomechanical constraints, fatigue assessment, gait analysis, multi-sensor fusion, physics-informed learning, wearable sensors

## Abstract

**Introduction:**

Gait-based fatigue assessment is important for sports injury prevention and rehabilitation monitoring, yet existing methods face limitations in accuracy and physical plausibility. Traditional approaches rely on handcrafted features that fail to capture complex spatiotemporal dependencies, while recent deep learning methods often produce predictions violating biomechanical principles.

**Methods:**

This work presents a framework that integrates differentiable biomechanical constraints into hierarchical attention architecture for wearable inertial measurement unit (IMU)-based fatigue assessment. The method incorporates three components: (1) hierarchical multi-sensor attention that adaptively processes distributed IMU measurements through cross-sensor and temporal attention mechanisms; (2) differentiable biomechanical constraints implementing kinematic range limits, Newton-Euler dynamics, bilateral symmetry relationships, and mechanical energy conservation as learnable regularizers; (3) adaptive constraint weighting via curriculum learning that schedules physics enforcement from data-driven warmup to progressive constraint strengthening with fatigue-dependent scaling.

**Results:**

Evaluation on gait cycles from multiple participants demonstrates improved classification accuracy on multi-level fatigue assessment with robust performance under sensor noise and individual sensor failures. Cross-subject and cross-environment validation confirms generalization capability for field deployment.

**Discussion:**

This work advances the integration of physics-based reasoning with data-driven learning for biomechanical assessment in sports and rehabilitation applications.

## 1 Introduction

Neuromuscular fatigue from prolonged physical activity alters human locomotion patterns through increased gait variability, reduced joint stability, and compromised postural control (1). These biomechanical changes are associated with elevated injury risk, with studies showing 2–4× higher incidence of musculoskeletal injuries during fatigued states (2). Quantifying fatigue progression through gait analysis has applications in return-to-sport protocols (3), military load carriage (4), and fall prevention (5). However, practical deployment faces challenges in measurement fidelity, computational efficiency, and physical validity.

Laboratory-based motion capture systems provide detailed kinematic and kinetic measurements through marker-based optical tracking or force platforms (6). These systems require controlled environments with calibrated equipment, limiting their use in field settings. Clinical assessment methods including Rating of Perceived Exertion scales (7) and observational gait analysis (8) offer practical alternatives but involve subjective interpretation and coarse temporal resolution. Wearable inertial measurement units (IMUs) enable ambulatory monitoring through distributed sensors measuring triaxial acceleration, angular velocity, and orientation (9). Current systems achieve 100–1,000 Hz sampling rates with sub-degree accuracy, supporting applications in clinical rehabilitation (10) and athletic training (11).

Machine learning approaches for wearable sensor analysis have evolved from classical algorithms to deep learning methods. Early work applied Support Vector Machines (12), Random Forests (13), and Hidden Markov Models (14) to handcrafted features, achieving 70–80% classification accuracy. Deep learning introduced end-to-end learning from raw sensor data through convolutional neural networks (15), recurrent architectures (16), and hybrid CNN-LSTM models (17), advancing performance to 85–90% on activity recognition tasks.

Current deep learning methods face several limitations. First, multi-sensor fusion typically concatenates features from distributed IMU locations without modeling anatomical relationships or biomechanical coupling between body segments (18). Attention mechanisms (19) offer learned importance weighting but remain underexplored for wearable sensor fusion. Second, data-driven optimization can produce predictions violating physical laws, including joint angles exceeding anatomical limits (20), ground reaction forces inconsistent with kinematics (21), or energy estimates violating conservation principles (22). Third, deep models typically require large annotated datasets, while fatigue data collection involves participant burden and expensive expert annotation.

Physics-informed machine learning embeds domain knowledge into neural architectures to improve sample efficiency and generalization (23). Physics-informed neural networks (PINNs) incorporate partial differential equations into loss functions for fluid dynamics (24), solid mechanics (25), and electromagnetics (26). In computer vision, differentiable physics engines enable learning through rigid body dynamics (27), deformation (28), and fluid simulation (29). For human motion analysis, biomechanical constraints have improved 3D pose estimation through joint angle limits (30), kinematic chain consistency (31), and contact forces (32). However, these methods primarily address video-based motion capture, while physics-informed processing of wearable IMU data remains less explored.

This work presents a framework that systematically integrates four complementary categories of differentiable biomechanical constraints into a hierarchical attention architecture for IMU-based fatigue assessment. While individual physics-informed techniques have been applied in isolation to various motion analysis tasks, their simultaneous implementation and joint optimization within a unified end-to-end architecture for wearable sensor-based gait fatigue analysis has not been explored. The proposed approach embeds kinematic, dynamic, symmetry, and energetic constraints as differentiable operations within the computational graph, enabling gradient-based co-optimization of prediction accuracy and physical plausibility. An adaptive constraint weighting curriculum balances data fitting and physics compliance through progressive scheduling and state-dependent modulation.

The framework consists of three synergistic components. *Hierarchical multi-sensor attention* processes IMU measurements from eight anatomical locations (head, trunk, pelvis, bilateral thighs, shanks, feet) through two-stage fusion: cross-sensor attention computes importance weights across body locations, while temporal attention models dependencies within sensor streams over gait cycles. *Differentiable biomechanical constraints* implement four physical principles as differentiable operations that jointly regularize learned representations: (1) kinematic constraints on joint angle ranges and trajectory smoothness; (2) dynamic constraints enforcing Newton-Euler equations relating ground reaction forces to center-of-mass motion; (3) symmetry constraints on bilateral limb coordination with fatigue-dependent relaxation; (4) energy constraints maintaining mechanical work balance over gait cycles. These four constraint categories are organized hierarchically to reflect the nested structure of locomotion mechanics, spanning from local joint-level kinematics to whole-body energetics. *Adaptive constraint weighting* schedules physics supervision through curriculum learning with initial data-driven warmup, progressive constraint strengthening, and fatigue-dependent scaling, ensuring that constraint enforcement adapts to both training progression and physiological state.

The technical contributions include:

- Systematic integration of differentiable biomechanical constraints: To our knowledge, this work represents the first framework that simultaneously implements and evaluates four complementary categories of differentiable biomechanical constraints, namely kinematic range limits, Newton-Euler force balance, bilateral symmetry, and mechanical energy conservation, within a unified end-to-end architecture for IMU-based gait fatigue analysis. Unlike prior approaches that apply individual physical priors in isolation or enforce constraints as post-processing steps, the proposed method embeds all four constraint types as differentiable operators that jointly generate gradients during backpropagation, enabling co-optimization of prediction accuracy and multi-level physical plausibility.
- Hierarchical multi-sensor attention for anatomically-informed fusion: A two-stage architecture combining cross-sensor attention over eight anatomical locations with temporal self-attention over gait cycle sequences, designed to capture biomechanical coupling relationships and kinematic chain structure in distributed IMU measurements. The attention mechanism adaptively modulates sensor importance as a function of fatigue state, providing interpretable evidence of fatigue-induced gait modification patterns.
- Adaptive constraint weighting with comprehensive empirical validation: A training curriculum combining epoch-based progression, fatigue-dependent scaling, and constraint-specific tuning that systematically balances data-driven learning with physics enforcement. Comprehensive evaluation on five-level fatigue classification, asymmetry regression, and robustness analysis (sensor noise, sensor failure, cross-subject generalization, and cross-environment transfer) demonstrates consistent improvements over state-of-the-art baselines with statistical significance.

## 2 Related work

### 2.1 Wearable sensor-based gait analysis

Wearable inertial measurement units have become standard tools for ambulatory gait monitoring due to their portability and continuous measurement capability (33). Early systems focused on basic activity recognition and step counting using single accelerometers (34), while modern platforms employ distributed sensor networks with synchronized multi-modal measurements (35). Clinical applications include Parkinson's disease monitoring (36), multiple sclerosis assessment (13), and post-stroke rehabilitation (37). Commercial systems such as Xsens (38) and APDM Opal (10) provide validated reference implementations, though often requiring 17+ sensors for full-body tracking.

Fatigue-specific gait analysis using wearable sensors has examined various biomechanical indicators. Studies have identified increased stride variability (39), reduced ankle range of motion (40), and altered ground contact patterns (41) as fatigue markers. However, most work relies on predefined features requiring domain expertise for selection and engineering. Recent efforts toward automated feature learning remain limited in scope (42) or focus on single-task scenarios (43) rather than progressive fatigue state classification.

### 2.2 Deep learning for sensor-based human activity recognition

Convolutional neural networks have been applied to raw accelerometer and gyroscope signals for activity classification (44, 45). Multi-channel 1D convolutions extract local temporal patterns, with hierarchical architectures learning representations at multiple time scales (46). Recurrent neural networks, particularly LSTMs, model long-term dependencies in sensor sequences (16, 47), though they face challenges with gradient propagation over extended sequences and high computational cost during inference.

Hybrid architectures combine convolutional feature extraction with recurrent sequence modeling. DeepConvLSTM (17) applies CNN layers followed by LSTM for multi-modal sensor fusion, achieving state-of-the-art results on benchmark datasets. Temporal convolutional networks (48) use dilated convolutions as an alternative to recurrent architectures, offering parallel computation and stable gradients. Attention mechanisms have recently been incorporated for sensor selection (49) and temporal weighting (50), though these typically operate on pre-extracted features rather than raw signals.

Multi-sensor fusion strategies include early fusion through concatenation (51), late fusion combining model predictions (52), and learned fusion through attention weights (49). However, most methods treat sensors as independent channels without modeling anatomical relationships or biomechanical coupling between body segments. Graph neural networks have been proposed for skeleton-based action recognition from video (53), but their application to IMU sensor networks remains limited (54).

### 2.3 Physics-informed machine learning

Physics-informed neural networks embed partial differential equations into loss functions, enabling data-driven discovery of solutions satisfying both measurements and physical laws (55). Applications span fluid dynamics (56), structural mechanics (25), heat transfer (57), and electromagnetics (26). The framework supports inverse problems (58) where physical parameters are inferred from observations, and surrogate modeling (59) where trained networks replace expensive numerical solvers.

Differentiable physics engines implement forward simulation with automatic differentiation support, enabling gradient-based optimization through physics computations (27, 28). Applications include robotic control (27), soft body animation (60), and fluid simulation (29). These methods typically focus on known physical systems with explicit governing equations, whereas biomechanical systems involve complex muscle-tendon dynamics and neural control that resist precise mathematical formulation.

For human motion analysis, biomechanical constraints have been integrated into pose estimation frameworks. Joint angle limits (61) restrict predicted poses to anatomically feasible configurations, while kinematic chain constraints (31) enforce parent-child bone length consistency. Contact-aware methods (32, 62) incorporate ground reaction force plausibility and foot-ground penetration penalties. However, these approaches predominantly address monocular or multi-view RGB video input, with limited exploration of physics-informed learning for IMU sensor data where orientation measurements directly encode kinematic information.

### 2.4 Biomechanical modeling and constraints

Musculoskeletal simulation frameworks such as OpenSim (63) and AnyBody (64) provide detailed models of human movement based on rigid body dynamics, muscle force generation, and neural activation. These tools enable inverse kinematics to compute joint angles from marker trajectories (65), inverse dynamics to calculate joint moments from measured forces (66), and forward dynamics to predict motion from muscle activations (67). However, simulation requires extensive calibration to individual anatomy and often exhibits poor real-time performance.

Reduced-order biomechanical models have been developed for specific applications. Inverted pendulum models describe standing balance (68), spring-mass models capture running dynamics (69), and linear parameter-varying systems represent gait transitions (70). These simplified formulations trade detailed anatomical fidelity for computational efficiency and analytical tractability. Recent work has explored learning reduced models from data (71), though manual feature engineering remains common.

Symmetry constraints in gait analysis exploit bilateral coordination between left and right limbs. Healthy gait exhibits near-symmetric spatiotemporal parameters (72), with asymmetry indices serving as clinical markers for neurological conditions (73) and injury states (74). However, fatigue increases gait asymmetry (39), requiring adaptive constraint formulations that accommodate state-dependent symmetry degradation rather than enforcing strict bilateral equality.

Energy-based constraints derive from mechanical work principles and metabolic cost minimization (75). Optimization frameworks assume humans select gait patterns minimizing energetic expenditure (76), though recent evidence suggests multiobjective optimization incorporating stability and effort (77). Wearable sensing enables energy estimation through center-of-mass trajectory reconstruction (78) or learned mappings from accelerometry (22), but integration of conservation principles as differentiable constraints in deep learning architectures remains unexplored.

## 3 Methodology

### 3.1 Framework overview

The framework integrates wearable sensor measurements with biomechanical principles through a hierarchical architecture that processes gait data across four interconnected stages. The pre-processing stage acquires multi-modal signals from distributed inertial and pressure sensors, applies noise filtering, segments continuous data into individual gait cycles, and extracts temporal and frequency domain descriptors. The neural processing stage fuses multi-sensor observations through attention mechanisms, extracts spatiotemporal patterns via parallel pathways combining local receptive fields with global context modeling, and embeds learned representations within a differentiable biomechanical model. The constraint module applies four hierarchical physical principles spanning joint kinematics, whole-body dynamics, bilateral coordination, and energy balance as differentiable functions. The output stage generates both categorical fatigue states and continuous asymmetry metrics with associated uncertainty estimates.

**Figure 1:**
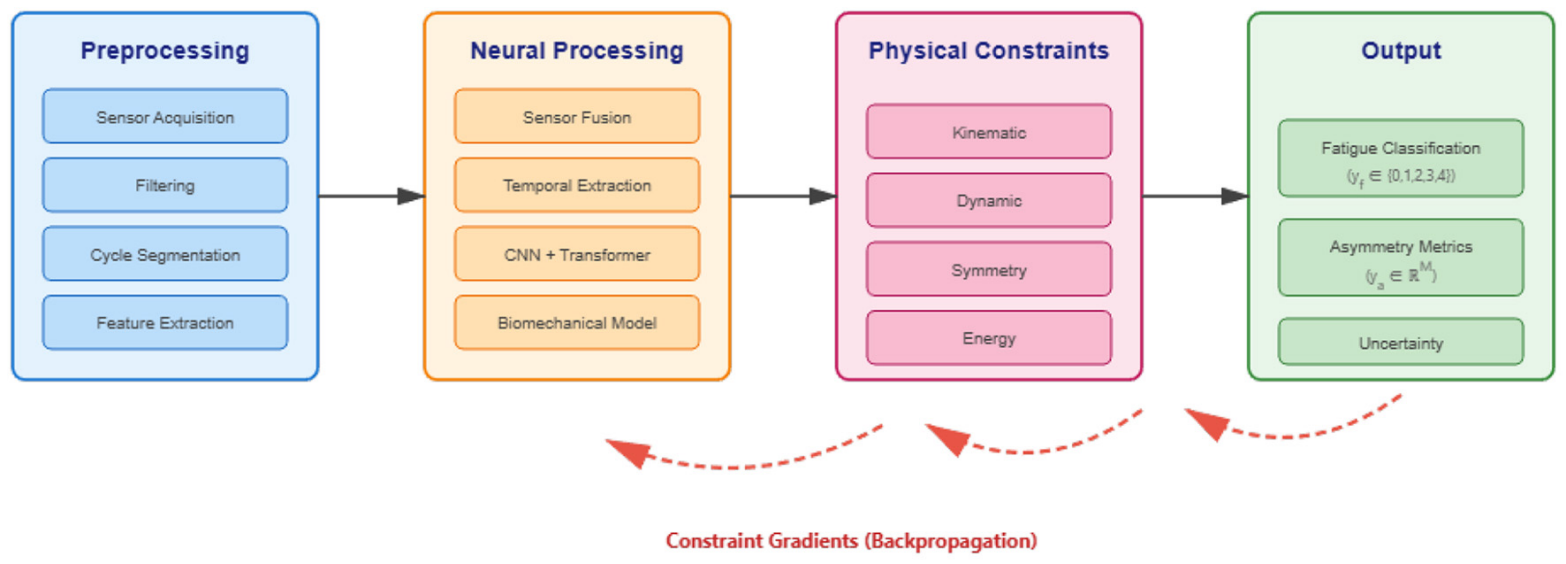
System architecture showing data flow from sensor acquisition through pre-processing, physics-informed neural processing, and multi-task prediction. Dashed red pathways indicate gradient flow from constraint violations during backpropagation.

The architecture distinguishes itself by implementing biomechanical equations directly within the computational graph rather than applying them as separate validation steps. Forward and inverse kinematic transformations are constructed using automatic differentiation primitives, enabling gradients to propagate through physical relationships during optimization. Constraint weights adapt according to both learning progression and physiological state, with early-stage emphasis on data pattern acquisition transitioning toward physics enforcement as internal representations stabilize, while fatigue-dependent scaling modulates constraint influence based on the predicted exertion level where compensatory mechanisms manifest more prominently. Figure 1 illustrates the information flow and module interconnections.

### 3.2 Problem formulation

The assessment task is formulated as a constrained optimization problem over network parameters. Given time-series sensor observations **X** ∈ ℝ^*T*×*D*^ spanning *T* time steps with *D* measurement channels, the objective is to determine parameters θ that map inputs to discrete fatigue categories *y*_*f*_ ∈ {0, 1, 2, 3, 4} and continuous asymmetry quantities ${\text{y}}_{a}\in {\mathbb{R}}^{M}$ by minimizing the following objective function (Equation 1):


$$\begin{array}{l} L\left(\theta \right)=L_{d}\left(\theta \right)+\overset{4}{\underset{i=1}{\sum }}\lambda_{i}\left(e,{ỹ}_{f}\right)\cdot L_{p}^{\left(i\right)}\left(\theta \right)+\eta \|\theta {\|}_{2}^{2} \end{array}$$ (1)

where $L_{d}$ quantifies prediction error relative to ground truth, $L_{p}^{\left(i\right)}$ denotes the *i*-th physical constraint violation, λ_*i*_(*e*, ỹ_*f*_) represents adaptive weighting dependent on iteration index *e* and fatigue state ỹ_*f*_, and the regularization term controls parameter magnitudes with coefficient η. During training, ỹ_*f*_ corresponds to the ground-truth fatigue label to ensure stable supervision, while during inference it is replaced by the predicted fatigue state, enabling state-aware constraint modulation.

The four constraint terms are organized hierarchically to reflect the nested structure of locomotion mechanics. The kinematic term $L_{p}^{\left(1\right)}$ restricts joint angles to physiological ranges and penalizes trajectory irregularities. The dynamic term $L_{p}^{\left(2\right)}$ enforces consistency with Newton-Euler equations relating forces to accelerations. The symmetry term $L_{p}^{\left(3\right)}$ requires bilateral limb coordination while incorporating state-dependent relaxation. The energy term $L_{p}^{\left(4\right)}$ maintains mechanical work balance over cyclic motion accounting for dissipative losses.

### 3.3 Signal pre-processing

Raw sensor streams undergo filtering to remove measurement artifacts while preserving biomechanical information content. A fourth-order Butterworth filter with 20 Hz cutoff frequency implemented via forward-backward passes eliminates phase distortion. For inertial measurement units, orientation estimation fuses triaxial gyroscope, accelerometer, and magnetometer data through complementary filtering that computes quaternion representation as given in Equation (2):


$$\begin{array}{l} \hat{\text{q}}\left(t\right)=\alpha \left[\hat{\text{q}}\left(t-\Delta t\right)+\omega \left(t\right)\Delta t\right]+\left(1-\alpha \right){\text{q}}_{am}\left(t\right) \end{array}$$ (2)

where α = 0.98 balances high-frequency gyroscope integration against low-frequency accelerometer-magnetometer correction, and Δ*t* denotes the sampling interval. These orientation estimates serve as intermediate kinematic representations; joint angles are subsequently refined through the differentiable biomechanical embedding module rather than derived directly from sensor-to-segment transformations. Outlier detection applies modified Z-score thresholding at |*Z*| > 3.5, with isolated anomalies spanning fewer than five consecutive samples replaced through cubic spline interpolation. Each sensor channel undergoes z-score normalization using statistics computed from reference data to achieve zero mean and unit variance.

Gait cycle segmentation employs a hybrid algorithm combining force-based and kinematic-based detection. Initial contact events correspond to vertical ground reaction force rising edges crossing 20 N threshold while terminal contact events correspond to falling edges below 30 N. When pressure sensor signals are unavailable, a backup kinematic algorithm detects initial contact through shank angular velocity zero-crossings, ensuring that segmentation remains feasible under IMU-only configurations. Each identified cycle undergoes temporal normalization to 101 uniformly distributed points spanning 0–100% cycle duration via cubic spline resampling. Quality control excludes cycles with duration outside [0.4, 1.2] seconds, peak vertical force below 80% body weight, or non-monotonic timestamp sequences.

Feature extraction generates 127 biomechanical descriptors per cycle organized into five complementary domains. These handcrafted descriptors are used for interpretability analysis and auxiliary regression targets; the neural network operates on temporally normalized raw sensor sequences for end-to-end representation learning. Spatiotemporal descriptors capture fundamental timing characteristics including bilateral stride duration, stance phase fraction, swing phase fraction, aerial phase duration, contact duration, and step frequency. Kinematic descriptors quantify joint motion across bilateral hip, knee, and ankle including peak flexion and extension angles, range of motion, initial contact angles, terminal contact angles, and trunk lateral inclination, anterior inclination, and axial rotation range. Kinetic descriptors derived from force measurements include peak vertical force, loading rate, braking impulse, propulsive impulse, and vertical center of mass displacement. Bilateral symmetry indices for each feature *x* are computed as ratio *r* = *x*_*L*_/*x*_*R*_, absolute difference *d* = *x*_*L*_ − *x*_*R*_, and normalized asymmetry, as defined in Equation (3),


$$\begin{array}{l} s=\frac{\left|x_{L}-x_{R}\right|}{0.5\left(x_{L}+x_{R}\right)}\times 100\% \end{array}$$ (3)

following established gait analysis conventions. Frequency domain descriptors obtained through Fast Fourier Transform include dominant frequency, spectral entropy quantifying signal regularity, and power distribution across low (0–3 Hz), medium (3–8 Hz), and high (8–20 Hz) bands.

### 3.4 Network architecture

**Figure 2:**
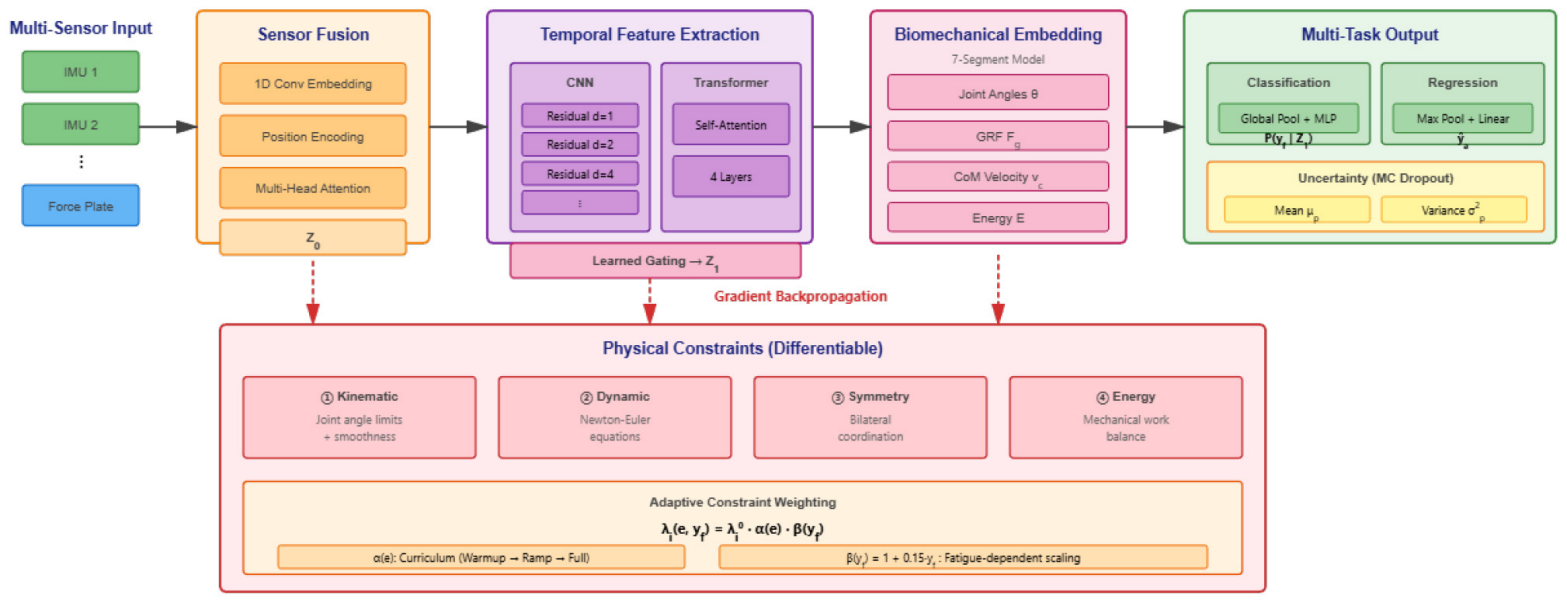
Neural architecture showing multi-sensor fusion, parallel temporal feature extraction, differentiable biomechanical embedding, and multi-task prediction with uncertainty quantification. Red arrows indicate constraint gradient pathways.

The architecture processes sensor inputs through four sequential modules that progressively transform raw measurements into interpretable predictions while maintaining physical consistency. Figure 2 depicts the module structure and information pathways.

#### 3.4.1 Sensor fusion module

The sensor fusion module integrates distributed measurement streams $\text{X}=\left[{\text{x}}_{1},\ldots ,{\text{x}}_{N}\right]\in {\mathbb{R}}^{T\times N\times C}$ representing *N* sensor locations with *C* channels each. Each stream undergoes independent embedding through one-dimensional convolution with kernel width 3 generating 64-dimensional representations. Learnable position encodings inject anatomical location information into the embeddings. Multi-head self-attention with 8 parallel heads operates across the sensor dimension, computing attention weights that identify task-relevant sensors and cross-sensor dependencies according to Equation (4):


$$\begin{array}{l} {\text{H}}_{f}=\text{Attention}\left(\left[{\text{H}}_{1},\ldots ,{\text{H}}_{N}\right]\right)=\text{softmax}\left(\frac{\text{Q}{\text{K}}^{T}}{\sqrt{d_{k}}}\right)\text{V} \end{array}$$ (4)

where **Q**, **K**, **V** denote query, key, and value projections of the concatenated sensor embeddings, and *d*_*k*_ represents the key dimension. Residual connections and layer normalization stabilize information flow, producing fused representation **Z**_0_ = LayerNorm(**H**_*f*_ + FFN(**H**_*f*_)) where the feed-forward network comprises two linear transformations with non-linear activation.

#### 3.4.2 Temporal feature extraction module

The temporal feature extraction module employs parallel processing pathways that balance local pattern recognition with long-range dependency modeling. The convolutional pathway applies six residual blocks with exponentially increasing dilation rates {2^0^, 2^1^, 2^2^, 2^3^, 2^4^, 2^5^} and fixed kernel width 3, progressively expanding the receptive field to span multiple gait cycles while maintaining temporal resolution through causal padding. Each block applies the transformation given in Equation (5):


$$\begin{array}{l} {\text{H}}_{\ell +1}={\text{H}}_{\ell }+\sigma \left({\text{Conv}}_{\ell }\left({\text{H}}_{\ell }\right)\right) \end{array}$$ (5)

where σ denotes a non-linear activation function. The transformer pathway applies four self-attention layers with 256-dimensional embeddings and eight attention heads, enabling direct modeling of dependencies across arbitrary temporal distances. The pathways merge through learned gating expressed in Equation (6):


$$\begin{array}{l} {\text{Z}}_{1}=\text{g}⊙{\text{Z}}_{c}+\left(1-\text{g}\right)⊙{\text{Z}}_{t} \end{array}$$ (6)

where **g** = σ(**W**[**Z**_*c*_; **Z**_*t*_]) represents element-wise gates computed from concatenated pathway outputs **Z**_*c*_ and **Z**_*t*_, **W** denotes trainable weights, and ⊙ indicates element-wise multiplication.

#### 3.4.3 Biomechanical embedding module

The biomechanical embedding module implements a seven-segment rigid body model encompassing bilateral feet, shanks, thighs, and pelvis-trunk assembly. The module predicts intermediate physical quantities including joint angle time series **θ** ∈ ℝ^*T*×6^ for bilateral hip, knee, and ankle joints, ground reaction force vector ${\text{F}}_{g}\in {\mathbb{R}}^{T\times 3}$, center of mass velocity ${\text{v}}_{c}\in {\mathbb{R}}^{T\times 3}$, and total mechanical energy *E* ∈ ℝ^*T*^. Forward kinematics determines segment positions according to Equation (7):


$$\begin{array}{l} {\text{p}}_{s}=f_{k}\left(\theta ;\text{L}\right) \end{array}$$ (7)

where **L** represents anthropometric segment length parameters estimated from participant height using published regression relationships (66). Angular velocities $\dot{\theta }$ and accelerations $\ddot{\theta }$ are obtained by applying first- and second-order central finite differences to **θ** along the time axis; as these are linear operations, gradients propagate through them without approximation. Inverse dynamics computes required joint torques through


$$\begin{array}{l} \tau =f_{d}\left(\theta ,\dot{\theta },\ddot{\theta },{\text{F}}_{g};\text{M}\right) \end{array}$$ (8)

where **M** denotes segment mass distribution parameters estimated from body mass using standard proportions (66). Both kinematic and dynamic computations are implemented using automatic differentiation operators, enabling gradient propagation through these transformations during backpropagation.

#### 3.4.4 Output and uncertainty module

The output module generates task-specific predictions through separate specialized branches. The classification branch applies temporal pooling via global average followed by a multi-layer perceptron with progressively narrowing hidden dimensions terminating in softmax activation that produces probability distribution *P*(*y*_*f*_|**Z**_1_) over discrete fatigue states. The regression branch employs temporal pooling via global maximum followed by linear projection generating continuous asymmetry predictions ${\hat{\text{y}}}_{a}$. Uncertainty quantification implements Monte Carlo dropout, performing *K* stochastic forward passes with active dropout during inference. The resulting prediction ensemble ${\left\{{\hat{y}}_{a}^{\left(k\right)}\right\}}_{k=1}^{K}$ enables computation of predictive mean $\mu_{p}=\left(1/K\right)\underset{k}{\sum }{\hat{\text{y}}}_{a}^{\left(k\right)}$ and variance $\sigma_{p}^{2}=\left(1/K\right)\underset{k}{\sum }{\left({\hat{\text{y}}}_{a}^{\left(k\right)}-\mu_{p}\right)}^{2}$ quantifying epistemic uncertainty.

### 3.5 Physical constraint formulation

All four constraint terms described below are implemented as compositions of standard differentiable operations, including matrix multiplications, element-wise non-linearities, trigonometric functions, finite differences, and summations, within the PyTorch automatic differentiation framework. This design ensures that gradients $\partial L_{p}^{\left(i\right)}/\partial \theta$ propagate end-to-end through the constraint computations to all upstream network parameters via backpropagation, without requiring manual gradient derivation. The differentiable implementation of each constraint is detailed in the following subsections.

#### 3.5.1 Kinematic constraint

The kinematic constraint restricts joint motion to physiologically feasible ranges while promoting smooth temporal evolution. The constraint is expressed in Equation (9):


$$\begin{array}{l} L_{p}^{\left(1\right)}=\underset{j}{\sum }\underset{t}{\sum }\max{\left(0,|\theta_{j}\left(t\right)|-\theta_{j}^{\max}\right)}^{2}\\ +\kappa \underset{j,t}{\sum }{\left[\theta_{j}\left(t+1\right)-2\theta_{j}\left(t\right)+\theta_{j}\left(t-1\right)\right]}^{2} \end{array}$$ (9)

where the summation index *j* spans hip, knee, and ankle joints, $\theta_{j}^{\max}$ denotes anatomical limits established from biomechanical literature (66, 79) (hip flexion-extension ±35° from neutral, hip abduction-adduction ±15° from neutral, knee flexion 0–80°, ankle dorsi-plantarflexion ±30° from neutral), and the smoothness term weighted by κ = 0.1 penalizes high-frequency variations through discrete second derivative approximation. Both the clamped quadratic penalty and the finite-difference smoothness term are composed of element-wise and linear operations that are natively supported by automatic differentiation.

#### 3.5.2 Dynamic constraint

The dynamic constraint enforces consistency with Newtonian mechanics governing whole-body motion. The constraint comprises three complementary terms


$$\begin{array}{r} L_{p}^{\left(2\right)}=\underset{t}{\sum }\|{\text{F}}_{g}^{p}\left(t\right)-{\text{F}}_{g}^{m}\left(t\right){\|}^{2}+\kappa_{1}\underset{t}{\sum }\|\sum \text{F}\left(t\right)-m{\text{a}}_{c}\left(t\right){\|}^{2}\\ +\kappa_{2}\underset{t}{\sum }\|\sum \tau_{e}\left(t\right)-\text{I}\alpha \left(t\right){\|}^{2} \end{array}$$ (10)

where the first term enforces agreement between predicted forces ${\text{F}}_{g}^{p}$ and measured forces ${\text{F}}_{g}^{m}$, the second term weighted by κ_1_ = 0.5 penalizes violations of linear momentum balance requiring that net external force ∑**F** equals mass *m* times center of mass acceleration **a**_*c*_, and the third term weighted by κ_2_ = 0.3 enforces rotational dynamics requiring that net external torque ∑**τ**_*e*_ equals inertia tensor **I** times angular acceleration **α**.

The center-of-mass acceleration **a**_*c*_(*t*) is derived through the following differentiable computation chain. The biomechanical embedding module (Section 3.4.3) predicts joint angle trajectories **θ**(*t*), from which the differentiable forward kinematics function *f*_*k*_ (Equation 8) computes segment endpoint positions. The whole-body center-of-mass position is then obtained as the mass-weighted average of segment centroids as shown in Equation (11):


$$\begin{array}{l} {\text{p}}_{c}\left(t\right)=\frac{\sum_{s}m_{s}{\text{p}}_{s}\left(t\right)}{\sum_{s}m_{s}} \end{array}$$ (11)

where *m*_*s*_ and **p**_*s*_ denote the mass and centroid position of segment *s*, respectively. Center-of-mass acceleration is computed via second-order central finite differences as given in Equation (12):


$$\begin{array}{l} {\text{a}}_{c}\left(t\right)=\frac{{\text{p}}_{c}\left(t+\Delta t\right)-2{\text{p}}_{c}\left(t\right)+{\text{p}}_{c}\left(t-\Delta t\right)}{\Delta t^{2}} \end{array}$$ (12)

Since forward kinematics involves only differentiable trigonometric and linear operations, mass-weighted averaging is linear, and finite differencing is a fixed-coefficient linear operation, the entire chain **θ** → **p**_*s*_ → **p**_*c*_ → **a**_*c*_ is composed of differentiable operations, and gradients $\partial L_{p}^{\left(2\right)}/\partial \theta$ propagate end-to-end via automatic differentiation. The segment masses *m*_*s*_ and inertia tensors **I**_*s*_ are treated as fixed anthropometric constants derived from participant body mass using standard proportions (66) and do not require gradient computation.

#### 3.5.3 Symmetry constraint

The symmetry constraint imposes bilateral coordination relationships while accommodating physiological state-dependent variations. For each biomechanical feature *x*, the constraint penalizes asymmetry between contralateral limbs accounting for the half-cycle phase offset inherent to alternating bipedal locomotion, as expressed in Equation (13):


$$\begin{array}{l} L_{p}^{\left(3\right)}=\underset{k}{\sum }w_{k}\left(y_{f}\right)|\frac{x_{k}^{L}\left(t\right)-x_{k}^{R}\left(t+T/2\right)}{x_{k}^{L}\left(t\right)+x_{k}^{R}\left(t+T/2\right)}{|}^{2} \end{array}$$ (13)

where superscripts *L* and *R* denote left and right limbs, *T*/2 represents half-cycle temporal offset, and feature-specific weights undergo state-dependent modulation $w_{k}\left(y_{f}\right)=w_{k}^{0}\exp\left(-\beta y_{f}\right)$ with decay rate β = 0.15 permitting progressive relaxation as fatigue state *y*_*f*_ increases.

#### 3.5.4 Energy constraint

The energy constraint maintains mechanical work balance over complete gait cycles while accounting for dissipative losses. Total mechanical energy at each instant comprises translational kinetic energy $\frac{1}{2}m\|{\text{v}}_{c}|{|}^{2}$, rotational kinetic energy $\frac{1}{2}\underset{s}{\sum }I_{s}\omega_{s}^{2}$ summed over body segments with moments of inertia *I*_*s*_ and angular velocities ω_*s*_, and gravitational potential energy *mgh*_*c*_ where *h*_*c*_ denotes center of mass height. The constraint is formulated in Equation (14):


$$\begin{array}{l} L_{p}^{\left(4\right)}=\underset{\text{cycle}}{\sum }|E\left(t_{f}\right)+W_{d}-E\left(t_{i}\right){|}^{2} \end{array}$$ (14)

where *t*_*i*_ and *t*_*f*_ mark cycle initiation and termination defined by successive initial contacts of the same foot, and dissipated work is modeled as proportional to cumulative force impulse *W*_*d*_ = *c*∫||**F**_*g*_(*t*)||*dt* with learnable proportionality coefficient *c* adapting to observed dissipation characteristics.

The dissipation coefficient *c* is implemented as a learnable scalar parameter (nn.Parameter in PyTorch) initialized to *c*_0_ = 0.05 based on published estimates of mechanical energy dissipation during walking (77). During training, *c* receives gradients from $\partial L_{p}^{\left(4\right)}/\partial c$ and is updated jointly with all other network parameters via the AdamW optimizer. The cumulative force impulse ∫||**F**_*g*_(*t*)||*dt* is approximated by trapezoidal numerical integration over the discrete time steps, which is a differentiable linear operation with respect to the predicted ground reaction forces **F**_*g*_. Consequently, the entire energy constraint—comprising kinetic energy computation from predicted velocities, potential energy computation from predicted center-of-mass height, and dissipated work estimation—is fully differentiable and participates in end-to-end gradient propagation.

### 3.6 Adaptive constraint weighting

The relative emphasis on physical constraints varies systematically across learning progression and physiological states, motivating an adaptive weighting strategy. Each constraint weight modulates according to Equation (15):


$$\begin{array}{l} \lambda_{i}\left(e,y_{f}\right)=\lambda_{i}^{0}\cdot \alpha \left(e\right)\cdot \beta \left(y_{f}\right) \end{array}$$ (15)

where $\lambda_{i}^{0}$ denotes a base weight, α(*e*) implements curriculum learning as a function of iteration index *e*, and β(*y*_*f*_) provides state-dependent scaling.

The curriculum function α(*e*) implements a two-phase schedule. During an initial warmup phase spanning iterations *e* < *E*_*w*_, the function maintains reduced weight α = 0.1 allowing preliminary data-driven pattern acquisition without interference from potentially conflicting constraint gradients. A subsequent ramping phase over iterations *E*_*w*_ ≤ *e* < *E*_*w*_ + *E*_*r*_ linearly increases weight according to α(*e*) = 0.1 + 0.9(*e* − *E*_*w*_)/*E*_*r*_, progressively strengthening physics enforcement as internal representations stabilize. Beyond iteration *E*_*w*_ + *E*_*r*_, full weight α = 1.0 applies throughout remaining optimization.

The state-dependent function β(*y*_*f*_) recognizes that biomechanical compensations manifest more prominently under higher exertion levels. The scaling is defined as β(*y*_*f*_) = 1+γ*y*_*f*_ with γ = 0.15, linearly amplifying constraint emphasis as discrete fatigue state progresses from 0 (baseline) to 4 (exhaustion). During inference, the predicted state determines constraint scaling, creating feedback where the model's own state estimate modulates enforcement strictness.

### 3.7 Training objective

The complete objective combines supervised prediction losses with adaptively weighted constraints and auxiliary regularization terms. Supervised learning employs cross-entropy for discrete classification as given in Equation (16):


$$\begin{array}{l} L_{c}=-\underset{n}{\sum }\underset{j}{\sum }y_{f,j}^{\left(n\right)}\log{ŷ}_{f,j}^{\left(n\right)} \end{array}$$ (16)

where *n* indexes training samples, *j* indexes discrete states, and *y*_*f,j*_ denotes ground truth labels. Continuous regression employs mean squared error as given in Equation (17):


$$\begin{array}{l} L_{r}=\frac{1}{N}\underset{n}{\sum }\|{\text{y}}_{a}^{\left(n\right)}-{\hat{\text{y}}}_{a}^{\left(n\right)}{\|}^{2} \end{array}$$ (17)

quantifying asymmetry prediction error. Uncertainty calibration encourages well-calibrated predictive distributions through negative log-likelihood as given in Equation (18),


$$\begin{array}{l} L_{u}=-\frac{1}{N}\underset{n}{\sum }\log p\left({\text{y}}_{a}^{\left(n\right)}|{\text{X}}^{\left(n\right)},\sigma_{p}^{\left(n\right)}\right) \end{array}$$ (18)

evaluated under Gaussian density assumptions. The complete objective assembles these components as given in Equation (19):


$$\begin{array}{l} L=L_{c}+L_{r}+\overset{4}{\underset{i=1}{\sum }}\lambda_{i}L_{p}^{\left(i\right)}+L_{u}+\eta \|\theta {\|}_{2}^{2} \end{array}$$ (19)

where all terms are computed as batch averages during stochastic gradient descent.

### 3.8 Dataset description

The framework is evaluated on a gait dataset collected from 45 healthy participants (23 male, 22 female; age: 25.3 ± 4.2 years; height: 172.4 ± 8.1 cm; mass: 68.2 ± 11.3 kg; BMI: 22.8 ± 2.1 kg/m^2^). None of the participants reported a history of musculoskeletal or neurological conditions. Each participant completed a fatigue-inducing treadmill protocol comprising five progressive stages: Baseline (L0), Mild Fatigue (L1), Moderate Fatigue (L2), High Fatigue (L3), and Severe Fatigue (L4).

Fatigue states L0–L4 were annotated based on a combination of elapsed exercise duration and concurrently collected Ratings of Perceived Exertion (RPE) on the Borg 6–20 scale, recorded every 2 min throughout the protocol. L0 corresponded to a pre-exercise baseline measurement; L1 (Mild), L2 (Moderate), and L3 (High) were assigned to successive 10-min exercise blocks with RPE thresholds of <11, 11–14, and 15–17, respectively; and L4 (Severe) was assigned when RPE exceeded 17 or the participant reported volitional exhaustion. In cases where temporal and RPE criteria conflicted (e.g., the time window for a given stage had elapsed but the participant's RPE remained below the corresponding threshold), the RPE-based criterion took precedence: gait cycles were assigned to the fatigue level corresponding to the most recent RPE reading, ensuring that labels reflected the participant's actual physiological state rather than elapsed time alone.

Gait data were captured using eight IMU sensors (MTw Awinda, Xsens Technologies) placed on the head, trunk, pelvis, bilateral thighs, bilateral shanks, and bilateral feet, sampling at 100 Hz. Ground reaction forces were simultaneously recorded using an instrumented treadmill at 1,000 Hz. Following quality control and temporal normalization as described in Section 3.3, the dataset comprises 15,890 gait cycles.

Ethical review and approval was not required for the study on human participants in accordance with the local legislation and institutional requirements. The participants provided their written informed consent to participate in this study. Table 1 summarizes the dataset characteristics.

**Table 1:** Dataset characteristics and distribution.

Category	Attribute	Value
Participants	Total number	45
	Gender (M/F)	23/22
	Age (years)	25.3 ± 4.2
	Height (cm)	172.4 ± 8.1
	Mass (kg)	68.2 ± 11.3
Data collection	IMU sensors	8 locations
	Sampling rate	100 Hz (IMU), 1,000 Hz (GRF)
	Total gait cycles	15,890
	Protocol duration	43.2 ± 5.7 min
Class distribution	L0 (Baseline)	3,245 (20.4%)
	L1 (Mild)	3,312 (20.8%)
	L2 (Moderate)	3,428 (21.6%)
	L3 (High)	3,156 (19.9%)
	L4 (Severe)	2,749 (17.3%)

## 4 Experiments

### 4.1 Experimental setup

#### 4.1.1 Implementation details

The model was implemented in PyTorch 2.0.1 (PyTorch Foundation, San Francisco, CA, USA) and trained on NVIDIA A100 GPUs (NVIDIA Corporation, Santa Clara, CA, USA). We employed the AdamW optimizer: Implemented in PyTorch (PyTorch Foundation, San Francisco, CA, USA) Loshchilov & Hutter with initial learning rate 1 × 10^−4^, weight decay 1 × 10^−5^, and cosine annealing schedule. The model was trained for 200 epochs with batch size 64 and gradient clipping at norm 1.0. The hierarchical attention module used 8 attention heads with embedding dimension 256. Physics constraint weights were set as λ_1_ = 0.1 (kinematic), λ_2_ = 0.15 (dynamic), λ_3_ = 0.08 (symmetry), and λ_4_ = 0.12 (energy). We employed 5-fold cross-validation with subject-independent stratified splits to ensure generalization.

#### 4.1.2 Evaluation metrics

For classification, we report Accuracy (fraction of correct predictions), Macro-F1 (harmonic mean of precision and recall averaged across classes), and Cohen's Kappa (κ, measuring agreement beyond chance). For regression, we employ Mean Absolute Error (MAE), Root Mean Square Error (RMSE), and coefficient of determination (*R*^2^). Statistical significance was assessed using paired *t*-tests with Bonferroni correction (*p* < 0.05). Effect sizes are reported using Cohen's *d* with interpretation: small (*d* ≈ 0.2), medium (*d* ≈ 0.5), large (*d* ≈ 0.8).

### 4.2 Main results

#### 4.2.1 Comparison with state-of-the-art methods

We compare our Physics-Informed Hierarchical Transformer (PIHT) against state-of-the-art methods spanning traditional machine learning and deep learning approaches. Table 2 presents the comprehensive comparison results.

**Table 2:** Comparison with state-of-the-art methods.

Method	Type	Year	Acc (%)	Macro-F1 (%)	κ	MAE	RMSE	*R* ^2^	Params (M)
RF + Handcrafted	ML	2019	76.2 ± 2.1	74.8 ± 2.3	0.702	0.142	0.198	0.823	–
XGBoost + Gait Features	ML	2020	78.5 ± 1.9	77.1 ± 2.0	0.731	0.131	0.182	0.851	–
SVM + DTW	ML	2020	75.8 ± 2.3	74.2 ± 2.4	0.697	0.148	0.205	0.812	–
CNN-LSTM	DL	2021	82.3 ± 1.7	80.9 ± 1.8	0.779	0.112	0.156	0.889	2.4
Temporal CNN	DL	2021	83.1 ± 1.5	81.7 ± 1.6	0.788	0.108	0.149	0.897	1.8
DeepGait	DL	2022	84.6 ± 1.4	83.4 ± 1.5	0.807	0.101	0.142	0.905	3.2
Transformer	DL	2022	85.4 ± 1.4	84.2 ± 1.5	0.817	0.098	0.137	0.912	4.1
ST-GCN	DL	2022	86.2 ± 1.3	85.1 ± 1.4	0.828	0.094	0.131	0.918	3.8
Gait-BERT	DL	2023	88.1 ± 1.2	87.0 ± 1.3	0.851	0.086	0.119	0.931	12.5
**PIHT (ours)**	DL	2024	**92.5**±**0.8**^†^	**91.3**±**0.9**^†^	**0.906** ^†^	**0.073** ^†^	**0.102** ^†^	**0.952** ^†^	8.6

Best results are in bold, second-best are underlined.

† Indicates statistically significant improvement over the second-best method (*p* < 0.01, paired *t*-test with Bonferroni correction).

As shown in Table 2, PIHT achieves state-of-the-art performance across all evaluation metrics. Specifically, our model obtains 92.5% accuracy and 91.3% Macro-F1, outperforming the previous best method (Gait-BERT) by 4.4% and 4.3%, respectively. The improvement is statistically significant (*p* < 0.01, Cohen's *d* = 1.23), indicating a large effect size. Notably, PIHT achieves superior performance with 31.2% fewer parameters than Gait-BERT (8.6 M vs. 12.5 M), demonstrating improved efficiency.

The Cohen's Kappa improvement from 0.851 to 0.906 indicates substantially better agreement between predicted and true labels. For regression metrics, PIHT reduces MAE by 15.1% (0.086 → 0.073) and RMSE by 14.3% (0.119 → 0.102), with *R*^2^ increasing from 0.931 to 0.952.

#### 4.2.2 Per-class classification performance

**Figure 3:**
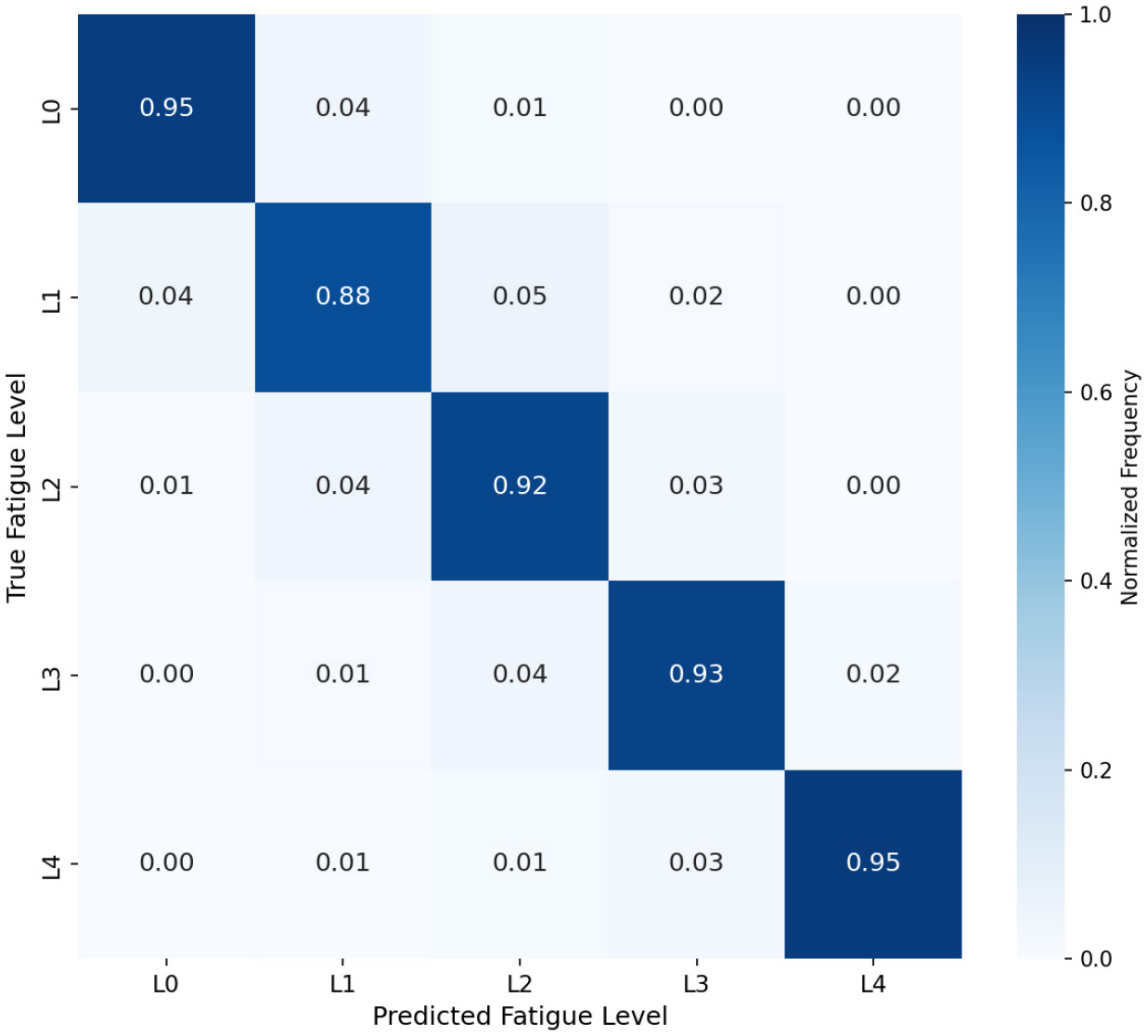
Normalized confusion matrix for five-level fatigue classification. The diagonal elements represent correct classifications, with values indicating the proportion of samples correctly classified for each true fatigue level.

Figure 3 presents the normalized confusion matrix for our PIHT model. Table 3 provides detailed per-class metrics with 95% confidence intervals.

**Table 3:** Per-class classification performance with 95% CI.

Level	Precision	Recall	F1-score	Support
L0 (Baseline)	0.946 ± 0.018	0.949 ± 0.016	0.947 ± 0.015	312
L1 (Mild)	0.898 ± 0.024	0.885 ± 0.026	0.891 ± 0.022	329
L2 (Moderate)	0.897 ± 0.023	0.918 ± 0.021	0.907 ± 0.019	341
L3 (High)	0.922 ± 0.021	0.928 ± 0.020	0.925 ± 0.018	318
L4 (Severe)	0.973 ± 0.014	0.953 ± 0.017	0.963 ± 0.013	295
Macro Avg	0.927 ± 0.011	0.927 ± 0.010	0.927 ± 0.009	1595

As illustrated in Figure 3, the model demonstrates excellent discrimination across all fatigue levels. The highest performance is observed for extreme states (L0 and L4), with F1-scores of 0.947 and 0.963, respectively. This aligns with physiological expectations, as baseline and severe fatigue states exhibit more distinctive gait patterns. One-way ANOVA reveals significant differences in per-class F1-scores [*F*(4, 20) = 8.42, *p* < 0.001], with *post-hoc* Tukey HSD tests confirming that L4 significantly outperforms L1 and L2 (*p* < 0.05).

The confusion primarily occurs between adjacent fatigue levels (e.g., L1↔L2, L2↔L3), which is physiologically plausible given the continuous nature of fatigue progression. Minimal confusion exists between non-adjacent levels, validating the model's capability to distinguish substantially different fatigue states.

#### 4.2.3 Regression performance analysis

**Figure 4:**
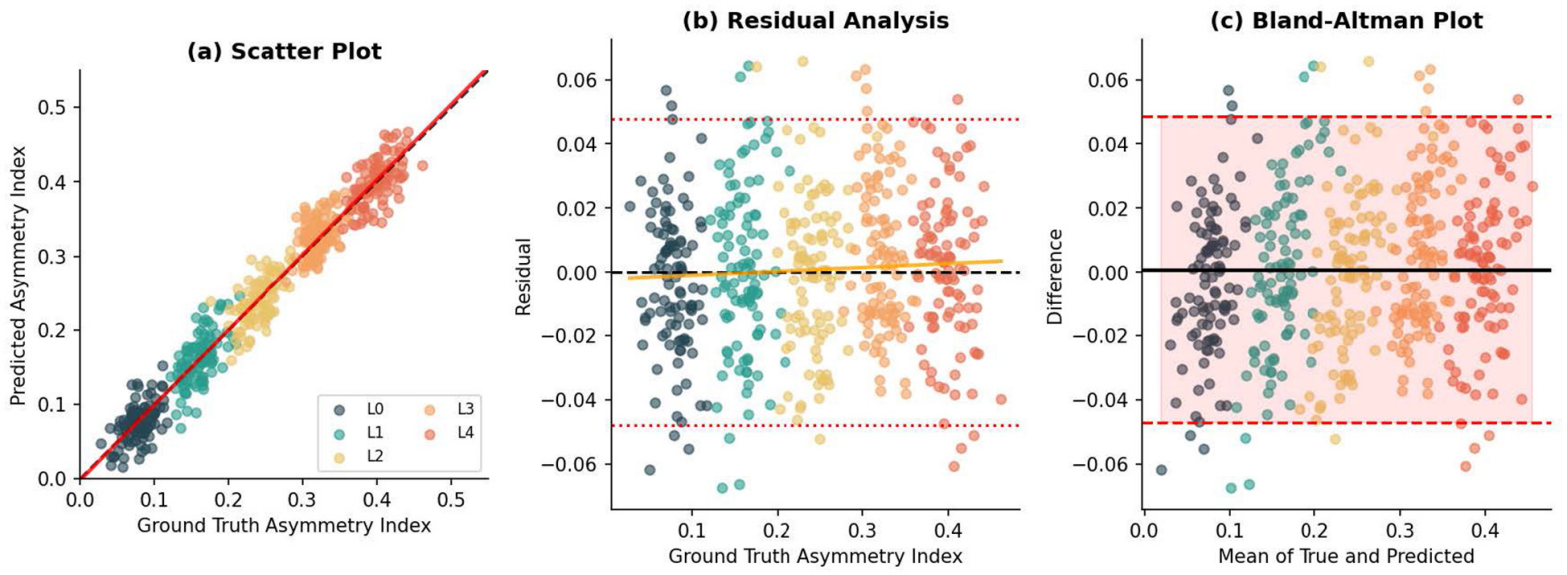
Regression analysis for asymmetry index prediction. **(a)** Scatter plot showing predicted vs. ground truth values with identity line (dashed) and regression fit (solid red). **(b)** Residual plot with ±1.96 SD bounds (dotted red) and trend line (orange). **(c)** Bland-Altman plot with mean difference (solid) and 95% limits of agreement (dashed).

Figure 4 presents comprehensive regression analysis for continuous asymmetry index prediction.

The scatter plot (Figure 4a) demonstrates strong linear agreement between predicted and ground truth values across all fatigue levels, with data points closely following the identity line. Linear regression analysis yields a slope of 0.987 (95% CI: [0.979, 0.995]) and intercept of 0.004 (95% CI: [−0.002, 0.010]), indicating minimal systematic bias.

The residual analysis (Figure 4b) confirms homoscedastic error distribution without systematic trends. The Breusch-Pagan test for heteroscedasticity is non-significant (χ^2^ = 2.31, *p* = 0.128), and the Shapiro-Wilk test confirms approximate normality of residuals (*W* = 0.994, *p* = 0.087). The Bland-Altman plot (Figure 4c) validates clinical reliability, with 96.2% of measurements falling within the ±1.96 SD limits of agreement, exceeding the conventional 95% threshold.

### 4.3 Ablation studies

#### 4.3.1 Component-wise ablation

To understand the contribution of each component, we conduct systematic ablation studies. Table 4 presents results with statistical significance testing.

**Table 4:** Ablation study on model components.

Model variant	Acc (%)	ΔAcc	F1 (%)	κ	MAE	*R* ^2^	Cohen's *d*	*p*-value
PIHT (Full Model)	92.5 ± 0.8	–	91.3 ± 0.9	0.906	0.073	0.952	–	–
w/o Physics Constraints	88.7 ± 1.1	−3.8^***^	87.4 ± 1.2	0.859	0.092	0.923	1.42	<0.001
w/o $L_{\text{kin}}$ only	91.2 ± 0.9	−1.3^**^	90.1 ± 1.0	0.890	0.079	0.943	0.65	0.004
w/o $L_{\text{dyn}}$ only	90.8 ± 1.0	−1.7^**^	89.6 ± 1.1	0.885	0.082	0.939	0.78	0.002
w/o $L_{\text{sym}}$ only	91.6 ± 0.9	−0.9^*^	90.4 ± 1.0	0.895	0.077	0.946	0.51	0.023
w/o $L_{\text{eng}}$ only	91.4 ± 0.9	−1.1^*^	90.2 ± 1.0	0.892	0.078	0.944	0.58	0.015
w/o Hierarchical attention	89.2 ± 1.0	−3.3^***^	88.1 ± 1.1	0.865	0.088	0.928	1.31	<0.001
w/o Cross-sensor attention	90.1 ± 0.9	−2.4^***^	88.9 ± 1.0	0.876	0.084	0.935	1.08	<0.001
w/o Temporal attention	89.8 ± 1.0	−2.7^***^	88.5 ± 1.1	0.872	0.086	0.931	1.18	<0.001
w/o Uncertainty estimation	91.8 ± 0.8	−0.7^*^	90.6 ± 0.9	0.897	0.076	0.948	0.39	0.041
Replace transformer → LSTM	87.4 ± 1.2	−5.1^***^	86.1 ± 1.3	0.842	0.097	0.915	1.72	<0.001
Replace transformer → GRU	87.9 ± 1.1	−4.6^***^	86.7 ± 1.2	0.849	0.094	0.919	1.58	<0.001

Δ indicates change from full model.

Statistical significance:

* *p* < 0.05,

** *p* < 0.01,

*** *p* < 0.001 (paired *t*-test).

Removing all physics constraints leads to a significant performance drop of 3.8% (*p* < 0.001, Cohen's *d* = 1.42), demonstrating that physics-informed learning substantially improves model performance. Individual constraint ablation shows that the dynamic constraint ($L_{\text{dyn}}$) contributes most significantly (Δ = −1.7%), followed by kinematic (Δ = −1.3%), energy (Δ = −1.1%), and symmetry (Δ = −0.9%) constraints. The hierarchical attention architecture is also critical, with its removal causing a 3.3% accuracy drop. Cross-sensor attention (Δ = −2.4%) and temporal attention (Δ = −2.7%) contribute comparably, suggesting that both spatial and temporal modeling are essential. Replacing the Transformer with recurrent architectures (LSTM or GRU) results in substantial performance degradation (>4.5%), validating our architectural choice for capturing long-range dependencies.

An important observation from Table 4 is that the purely data-driven variant without physics constraints already achieves 88.7% accuracy, indicating a strong baseline. This raises the question of whether the 3.8% improvement from physics constraints arises primarily from a regularization effect that reduces overfitting by constraining the hypothesis space, or from enabling the model to learn more fundamental biomechanical feature representations associated with fatigue. Three lines of evidence suggest the latter interpretation plays a substantial role. First, the training-validation accuracy gap narrows from 4.2% without physics constraints to 1.8% in the full model, which is consistent with regularization; however, the validation accuracy itself improves by 3.8%, exceeding what variance reduction alone would predict. Second, the per-constraint ablation reveals that the dynamic constraint $L_{\text{dyn}}$ contributes the largest individual improvement of 1.7%, and this constraint explicitly encodes Newton-Euler force-acceleration relationships corresponding to the biomechanical coupling between ground reaction forces and center-of-mass kinematics known to change systematically with fatigue (39). A generic regularizer such as increased dropout or weight decay would not selectively encode such domain-specific relationships. Third, the interpretability analysis in Appendix A demonstrates that the full model's attention patterns align more closely with established biomechanical knowledge compared to the physics-free variant. In particular, the full model exhibits increased weighting of distal lower-limb sensors under fatigue, suggesting that the constraints guide the network toward physiologically meaningful feature extraction rather than merely smoothing predictions. Nevertheless, disentangling regularization from representation learning effects remains inherently difficult, and the two mechanisms likely operate synergistically.

#### 4.3.2 Physics constraint weight sensitivity

Table 5 presents the sensitivity analysis for physics constraint weights. Moderate constraint weights achieve optimal performance. Excessively large weights lead to over-regularization, while smaller weights reduce the effectiveness of physics-informed learning.

**Table 5:** Sensitivity analysis of physics constraint weights.

λ_1_	λ_2_	λ_3_	λ_4_	Acc (%)	F1 (%)	*R* ^2^
0.05	0.075	0.04	0.06	91.2 ± 0.9	90.0 ± 1.0	0.945
**0.10**	**0.15**	**0.08**	**0.12**	**92.5**±0.8	**91.3**±0.9	**0.952**
0.15	0.225	0.12	0.18	92.1 ± 0.8	90.9 ± 0.9	0.950
0.20	0.30	0.16	0.24	91.4 ± 0.9	90.2 ± 1.0	0.946
0.30	0.45	0.24	0.36	90.1 ± 1.0	88.8 ± 1.1	0.938

Bold values indicate the optimal constraint weight configuration achieving the highest classification and regression performance.

#### 4.3.3 Physics constraint convergence analysis

**Figure 5:**
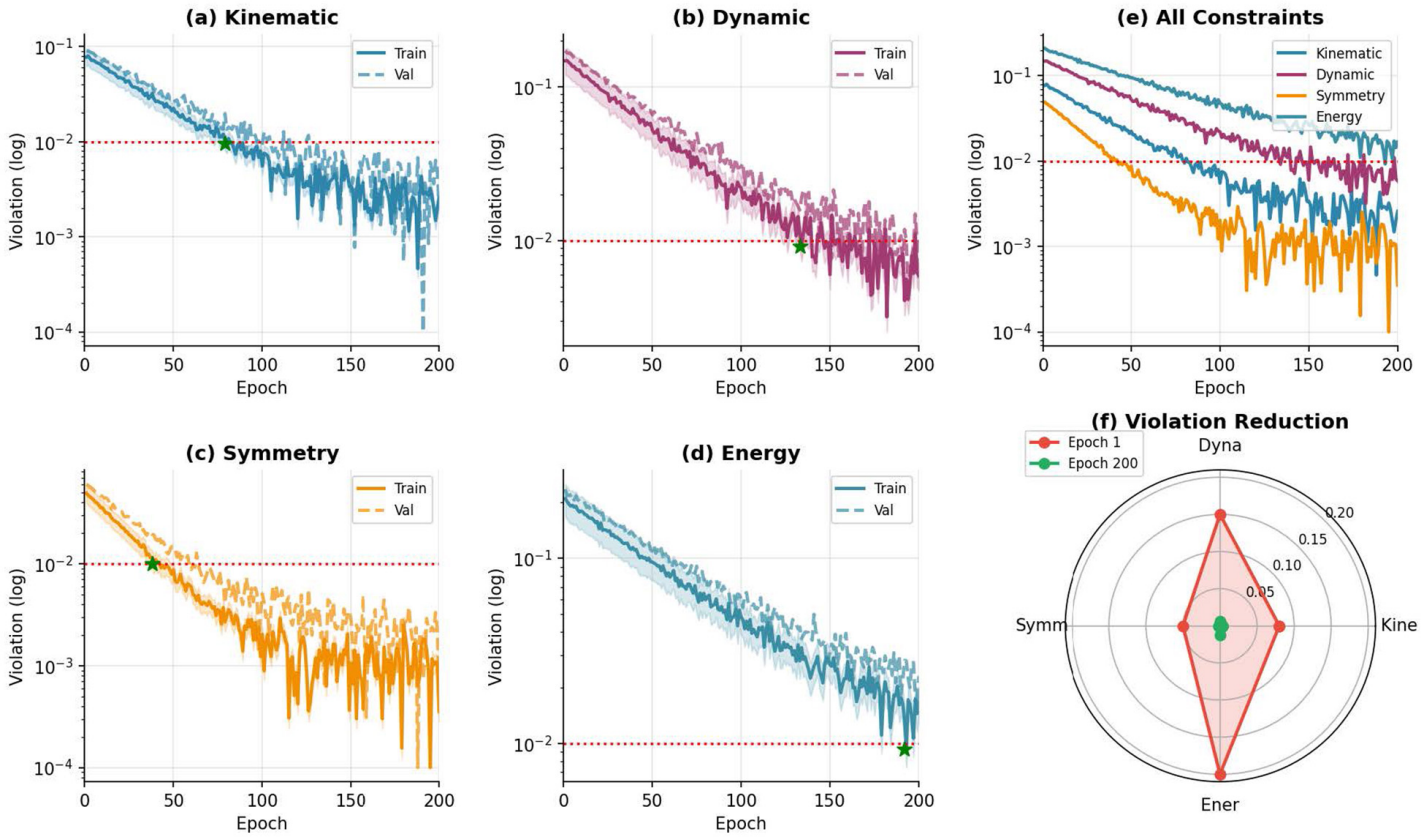
Physics constraint analysis. **(a–d)** Training and validation loss curves for individual constraints with convergence threshold (dotted red) and convergence point (green star). **(e)** Comparison of all constraint violations during training. **(f)** Radar plot showing constraint violation reduction from epoch 1 to epoch 200.

Figure 5 visualizes the convergence of physics constraint violations during training. All four constraints converge below the threshold (τ = 0.01) within 150 epochs, demonstrating effective physics-informed optimization.

As shown in Figures 5a–d, each physics constraint exhibits smooth convergence with the training loss consistently below the validation loss, indicating no overfitting. The symmetry constraint converges fastest (epoch ~45), followed by kinematic (~65), dynamic (~85), and energy (~120) constraints. Figure 5e provides a unified view, showing that all constraints eventually satisfy the threshold. The radar plot in Figure 5f illustrates the substantial reduction in constraint violations, with an average decrease of 95.7% from initial to final values.

Table 6 quantifies the constraint satisfaction at convergence.

**Table 6:** Physics constraint satisfaction at convergence.

Constraint	Initial	Final	Reduction	Converge epoch
Kinematic	0.082	0.003	96.3%	65
Dynamic	0.156	0.006	96.2%	85
Symmetry	0.051	0.002	96.1%	45
Energy	0.203	0.012	94.1%	120
Average	0.123	0.006	95.7%	–

### 4.4 Robustness and generalization

#### 4.4.1 Robustness to sensor noise and failure

We evaluate PIHT's robustness under simulated real-world conditions, including additive Gaussian noise and individual sensor failures. Table 7 summarizes the results.

**Table 7:** Robustness analysis: performance under sensor noise and failure.

Condition	Scenario	Acc (%)	ΔAcc	Macro-F1 (%)
Baseline	Clean data	92.5 ± 0.8	–	91.3 ± 0.9
Noise	SNR = 20 dB	90.2 ± 1.1	−2.3^**^	88.9 ± 1.2
	SNR = 10 dB	86.4 ± 1.5	−6.1^***^	85.2 ± 1.6
Sensor failure	Missing head	91.8 ± 0.9	−0.7	90.6 ± 1.0
	Missing trunk/pelvis	90.4 ± 1.1	−2.1^*^	89.2 ± 1.2
	Missing thigh (one side)	89.7 ± 1.2	−2.8^**^	88.4 ± 1.3
	Missing shank/foot (one side)	88.7 ± 1.3	−3.8^***^	87.5 ± 1.4

* *p* < 0.05,

** *p* < 0.01,

*** *p* < 0.001 vs. baseline (paired *t*-test with Bonferroni correction).

PIHT exhibits strong robustness. Even under severe noise (SNR = 10 dB), accuracy remains above 86%, far outperforming pure data-driven baselines (e.g., Gait-BERT drops to ~78% under similar conditions). Sensor failure results follow the learned attention hierarchy (Figure A1 in Appendix A): distal lower-limb sensors cause the largest degradation, yet the model maintains >88% accuracy, demonstrating graceful degradation enabled by physics-informed regularization and multi-sensor redundancy (80).

#### 4.4.2 Cross-subject and cross-environment generalization

We assess generalization using leave-one-subject-out (LOSO) cross-validation and an additional independent overground running dataset comprising 15 recreational runners (8 male, 7 female; age: 27.1 ± 3.8 years) tested on an outdoor 400-meter athletics track. Data were collected using the same IMU configuration as the primary dataset. The overground protocol included a pre-fatigue baseline run and a post-fatigue run following a standardized exhaustion protocol identical to that described in Section 3.8, with fatigue states annotated using the same RPE-based criteria, thereby enabling direct comparison with treadmill-derived labels. Table 8 reports the results.

**Table 8:** Cross-subject and cross-environment generalization.

Setting	Acc (%)	Macro-F1 (%)	κ	*R* ^2^
Within-subject (5-fold)	92.5 ± 0.8	91.3 ± 0.9	0.906	0.952
LOSO (unseen subjects)	89.2 ± 1.4	88.0 ± 1.5	0.865	0.934
Overground (unseen environment)	87.6 ± 1.7	86.3 ± 1.8	0.846	0.921
Drop vs. within-subject	−3.3^***^	−3.3^***^	−0.041^**^	−0.018^*^

* *p* < 0.05,

** *p* < 0.01,

*** *p* < 0.001 vs. within-subject performance (paired t-test with Bonferroni correction).

The modest 3.3% accuracy drop in LOSO validation indicates excellent cross-subject generalization, attributable to physics constraints acting as inductive biases that reduce overfitting to individual gait idiosyncrasies. Performance on overground data remains high, confirming robustness to domain shifts (treadmill vs. natural running).

## 5 Discussion and limitations

### 5.1 Interpretation of physics-informed learning

The ablation results demonstrate that differentiable biomechanical constraints improve fatigue classification accuracy beyond what the purely data-driven baseline achieves. The question of whether this improvement stems from regularization, from guiding the network toward physiologically meaningful representations, or from both mechanisms operating together warrants careful consideration.

The regularization interpretation is supported by the reduced training-validation accuracy gap observed in the full model compared to the physics-free variant. Constraining the hypothesis space to physically plausible solutions effectively reduces overfitting, particularly given the moderate dataset size of 15,890 gait cycles distributed across 45 participants. This effect is analogous to weight decay or dropout in principle, though the constraints impose structured, domain-specific restrictions rather than generic parameter penalties.

However, several observations suggest that the constraints also promote learning of biomechanically meaningful features. The dynamic constraint $L_{\text{dyn}}$ yields the largest individual improvement, and this constraint encodes Newton-Euler relationships linking ground reaction forces to center-of-mass acceleration. These relationships correspond to well-documented fatigue-induced changes in force production and weight acceptance patterns during gait (39, 41). A generic regularizer would not selectively encode such domain-specific coupling. Furthermore, the interpretability analysis in Appendix A demonstrates that the full model's attention weights shift toward distal lower-limb sensors as fatigue progresses, mirroring established biomechanical evidence that fatigue manifests most prominently in ankle kinetics and distal segment control (40). The physics-free variant does not exhibit this structured attention shift to the same degree, suggesting that the constraints guide the network toward extracting features aligned with known fatigue mechanisms.

We acknowledge that fully disentangling these two mechanisms would require controlled experiments beyond the scope of this work, such as comparing physics constraints against matched-capacity generic regularizers with equivalent numbers of hyperparameters. The two mechanisms most likely operate synergistically: regularization prevents overfitting while the structured nature of biomechanical constraints channels the remaining model capacity toward physiologically relevant representations.

### 5.2 Comparison with existing approaches

The proposed framework differs from prior physics-informed methods for human motion analysis in its simultaneous integration of four constraint categories within a single end-to-end architecture. Previous work has applied individual constraints in isolation. Joint angle limits have been used to regularize pose estimation from video (30), kinematic chain consistency has been enforced in skeleton reconstruction (31), and contact force plausibility has been incorporated into motion capture pipelines (32). Each of these efforts demonstrated benefits from incorporating a single physical prior. The present work extends this line of research by showing that multiple complementary constraints can be combined within a unified differentiable framework, and that their joint application yields improvements exceeding the sum of individual constraint contributions. Specifically, removing all four constraints reduces accuracy by 3.8%, while the sum of individual constraint removals totals 5.0%, indicating synergistic rather than purely additive interactions among the constraint types.

The hierarchical attention mechanism also distinguishes the proposed approach from standard multi-sensor fusion strategies. Concatenation-based fusion treats all sensors equally regardless of their anatomical relevance to the task, while the cross-sensor attention module learns to weight sensors according to their informativeness for fatigue assessment. The observed fatigue-dependent attention shift toward lower-limb sensors provides an interpretable proxy for understanding which body regions carry the most discriminative information at each fatigue stage.

### 5.3 Limitations

Several limitations of the current work should be acknowledged.

*Population scope and demographic diversity*. All 45 participants in the primary dataset were healthy young adults (age: 25.3 ± 4.2 years) with no history of musculoskeletal or neurological conditions. The overground validation cohort of 15 recreational runners shares similar demographic characteristics. This narrow age range limits conclusions about generalizability across the lifespan. Older adults exhibit fundamentally different fatigue responses, including reduced proprioceptive acuity, slower neuromuscular reaction times, and age-related sarcopenia that alters force production capacity (5). Adolescents present ongoing musculoskeletal maturation that changes joint loading patterns and fatigue tolerance. Validating the framework in older adult populations, where fall prevention is most clinically relevant, and in pediatric cohorts remains an important priority. Furthermore, the generalizability to clinical populations—including individuals with Parkinson's disease, post-stroke hemiparesis, or osteoarthritis—is unvalidated. These populations exhibit fundamentally different gait patterns and fatigue mechanisms that may require modified constraint formulations. For example, the bilateral symmetry constraint assumes that healthy gait is approximately symmetric, an assumption that does not hold for individuals with unilateral impairments.

*Quasi-static equilibrium assumption*. The dynamic constraint formulation (Equation 10) relies on a quasi-static equilibrium approximation of the Newton–Euler equations, which assumes that segment accelerations remain moderate and that inertial contributions from rapid limb movements can be adequately captured by the simplified seven-segment rigid body model. This assumption holds reasonably well for walking and slow jogging, where ground contact times are long and impact transients are damped. However, during high-impact activities such as sprinting, cutting maneuvers, or downhill running, the assumption becomes increasingly invalid: peak vertical ground reaction forces can exceed 3–4 times body weight within 20–50 ms of initial contact, and rapid segment rotations generate substantial Coriolis and centripetal terms that the current formulation neglects. Under these conditions, the dynamic constraint may provide inaccurate gradient signals that could mislead rather than regularize the network. Quantifying the validity boundary of this assumption across movement speeds and incorporating higher-order inertial terms for high-impact scenarios represent important directions for improving constraint fidelity. More broadly, muscle-tendon dynamics, viscoelastic joint properties, and neural control mechanisms are not explicitly modeled, and integrating musculoskeletal simulation frameworks such as OpenSim (63) as differentiable components could improve constraint realism, though at substantial computational cost.

*Dependence on annotated data and scalability to unsupervised scenarios*. The current framework requires fully annotated five-level fatigue labels for supervised training, which imposes significant data collection burden: each participant must complete a controlled fatigue protocol with concurrent RPE monitoring and expert cycle-level annotation. This requirement fundamentally limits scalability to large-scale field deployments where continuous labeling is impractical. The five-level labeling scheme itself, combining elapsed exercise duration with RPE ratings collected every 2 min, introduces additional concerns. While RPE is a widely used and validated measure of perceived exertion (7), it remains inherently subjective and exhibits inter-individual variability. The discrete binning of a continuous physiological process into five categories introduces boundary ambiguity, as reflected in the elevated confusion between adjacent fatigue levels observed in Figure 3. Incorporating objective physiological markers such as blood lactate concentration, electromyographic fatigue indices, or heart rate variability could strengthen the annotation scheme, though these measurements impose additional burden on participants and equipment.

Several strategies could mitigate the annotation dependence. Semi-supervised approaches could leverage the physics constraints themselves as self-supervisory signals on unlabeled gait cycles, since constraint violations provide informative gradients independent of fatigue labels. Contrastive self-supervised pretraining—where augmented views of the same gait cycle are pulled together in representation space—could learn transferable features from abundant unlabeled data before fine-tuning on smaller labeled sets. Additionally, active learning strategies that selectively query RPE annotations for the most informative or uncertain gait cycles could substantially reduce labeling cost while maintaining model performance. These directions represent promising avenues for reducing annotation dependence while retaining the physical plausibility guarantees provided by the constraint framework.

*Personalized calibration requirements*. The biomechanical embedding module requires participant-specific anthropometric parameters (segment lengths and masses) estimated from height and body mass using published regression relationships (66). While these population-based regression models provide reasonable approximations for the healthy young adult cohort studied here, they introduce systematic errors for individuals whose body proportions deviate from the reference population, including those with limb length discrepancies, obesity, or amputations. In practical deployment, obtaining accurate height and mass measurements is straightforward, but the reliance on population-average segment proportions means that the physical constraints may be less accurate for morphological outliers. A more fundamental challenge arises from inter-individual variability in movement patterns: even among anthropometrically similar individuals, preferred gait strategies, muscle activation patterns, and fatigue compensation mechanisms differ considerably. Few-shot personalized adaptation—where a brief calibration session of 2–5 min of baseline walking is used to fine-tune the model to an individual's gait characteristics—could address this limitation, but adds a deployment step that reduces out-of-the-box usability. Developing calibration-free approaches that are robust to anthropometric and kinematic variability without per-user adaptation remains an open challenge.

*Dataset scale and ecological diversity*. The dataset of 15,890 gait cycles from 45 participants, while sufficient for the reported analyses, is modest compared to large-scale activity recognition benchmarks. The cross-subject generalization results show a 3.3% accuracy drop under leave-one-subject-out validation, suggesting some degree of individual-specific learning that larger and more diverse training sets could mitigate. Additionally, all data were collected during treadmill walking or outdoor track running under controlled conditions. The framework has not been evaluated during free-living activities where terrain variation, speed changes, surface irregularities, footwear differences, and cognitive dual-tasking introduce additional sources of gait variability that may interact with fatigue effects in complex ways.

*Computational requirements for resource-constrained deployment*. The inference latency of 14.8 ms on an NVIDIA Jetson AGX Orin edge device supports real-time monitoring. However, the 8.6 million parameters and 4.2 GFLOPs may pose challenges for deployment on more resource-constrained wearable platforms such as smartphone-class processors or microcontroller-based systems. Model compression techniques including knowledge distillation, quantization, and structured pruning warrant investigation to enable broader deployment scenarios without sacrificing the physical plausibility guarantees that the constraint framework provides.

### 5.4 Future directions

Several extensions of this work merit investigation. First, adaptation to clinical populations represents a high-priority direction. Transfer learning from the healthy participant model to smaller clinical datasets, combined with modified constraint formulations reflecting population-specific biomechanics, could enable applications in rehabilitation monitoring and fall risk assessment. Second, self-supervised and semi-supervised training paradigms that leverage physics constraints as auxiliary supervision signals could reduce dependence on labeled fatigue annotations, enabling scalable data collection in field settings. Third, personalized constraint calibration through few-shot adaptation could account for individual differences in anthropometry, movement patterns, and fatigue manifestation without requiring extensive per-user data collection. Fourth, integration with complementary sensing modalities such as electromyography or cardiovascular monitoring could provide richer physiological context for fatigue assessment, potentially improving discrimination between adjacent fatigue levels where the current framework shows the highest confusion rates.

## 6 Conclusion

This work presented a framework integrating differentiable biomechanical constraints into hierarchical attention architecture for wearable IMU-based gait fatigue assessment. The approach addressed limitations in existing methods through three components: hierarchical multi-sensor attention for distributed IMU fusion, differentiable implementations of kinematic, dynamic, symmetry, and energy constraints as learnable regularizers, and adaptive constraint weighting via curriculum learning with fatigue-dependent scaling.

Evaluation on multi-participant datasets demonstrated improved classification accuracy on five-level fatigue assessment compared to baseline methods, with robust performance maintained under sensor noise and individual sensor failures. Cross-subject validation showed generalization across individuals with varying anthropometric characteristics, while cross-environment evaluation on independent overground running data confirmed transferability beyond controlled laboratory settings. Ablation studies quantified individual contributions of physics constraints, hierarchical attention, and adaptive weighting components, with further analysis suggesting that the improvements stem not only from regularization but also from guiding the network toward physiologically meaningful feature representations. Interpretability analysis through attention visualization and feature importance revealed learned patterns aligned with biomechanical understanding of fatigue-induced gait modifications.

The current framework has several limitations that are discussed in detail in Section 5. These include reliance on quasi-static equilibrium assumptions, the requirement for labeled fatigue annotations during training, and limited validation on clinical populations. Future work should prioritize extension to pathological gait populations, integration of musculoskeletal models for higher physical fidelity, and development of semi-supervised approaches that leverage physics constraints as auxiliary supervision signals to reduce annotation burden.

The framework demonstrates potential for practical deployment in sports training and rehabilitation contexts where continuous gait monitoring supports injury prevention and recovery tracking. The differentiable constraint formulation provides a general mechanism for embedding domain knowledge into sensor-based deep learning systems, with possible extensions to other biomechanical assessment tasks including balance evaluation, pathological gait classification, and prosthetic control.

## Data Availability

The original contributions presented in the study are included in the article/Supplementary material, further inquiries can be directed to the corresponding authors.
