# Supplementary figures and images for: Physics-informed hierarchical transformer for wearable sensor-based gait fatigue assessment
Source: Front Public Health. 2026 Apr 13;14:1794241. doi: 10.3389/fpubh.2026.1794241 (PMC13111241; doi:10.3389/fpubh.2026.1794241)

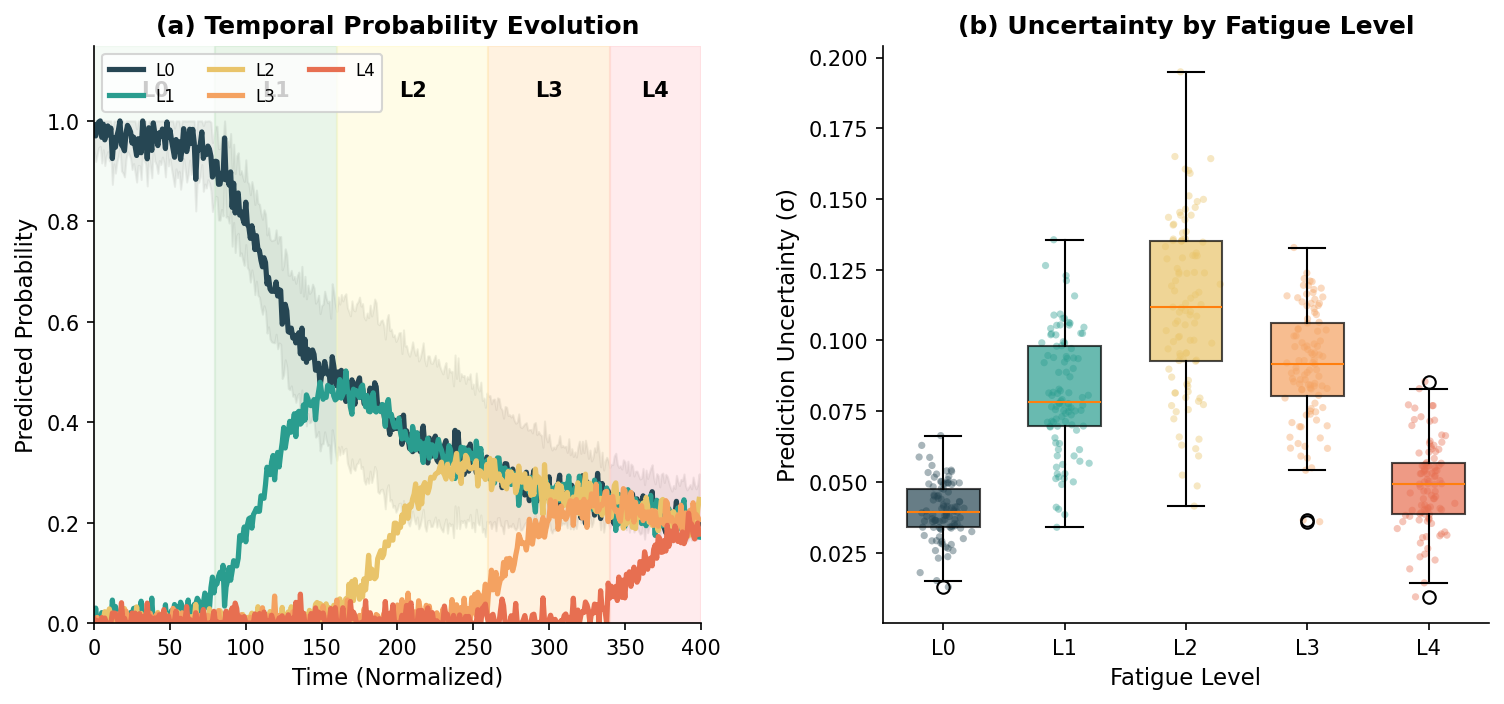

Supplement: Supplementary file 2 [file Supplementary_file_1.zip › Supplementary_Material/Figure8.jpg]

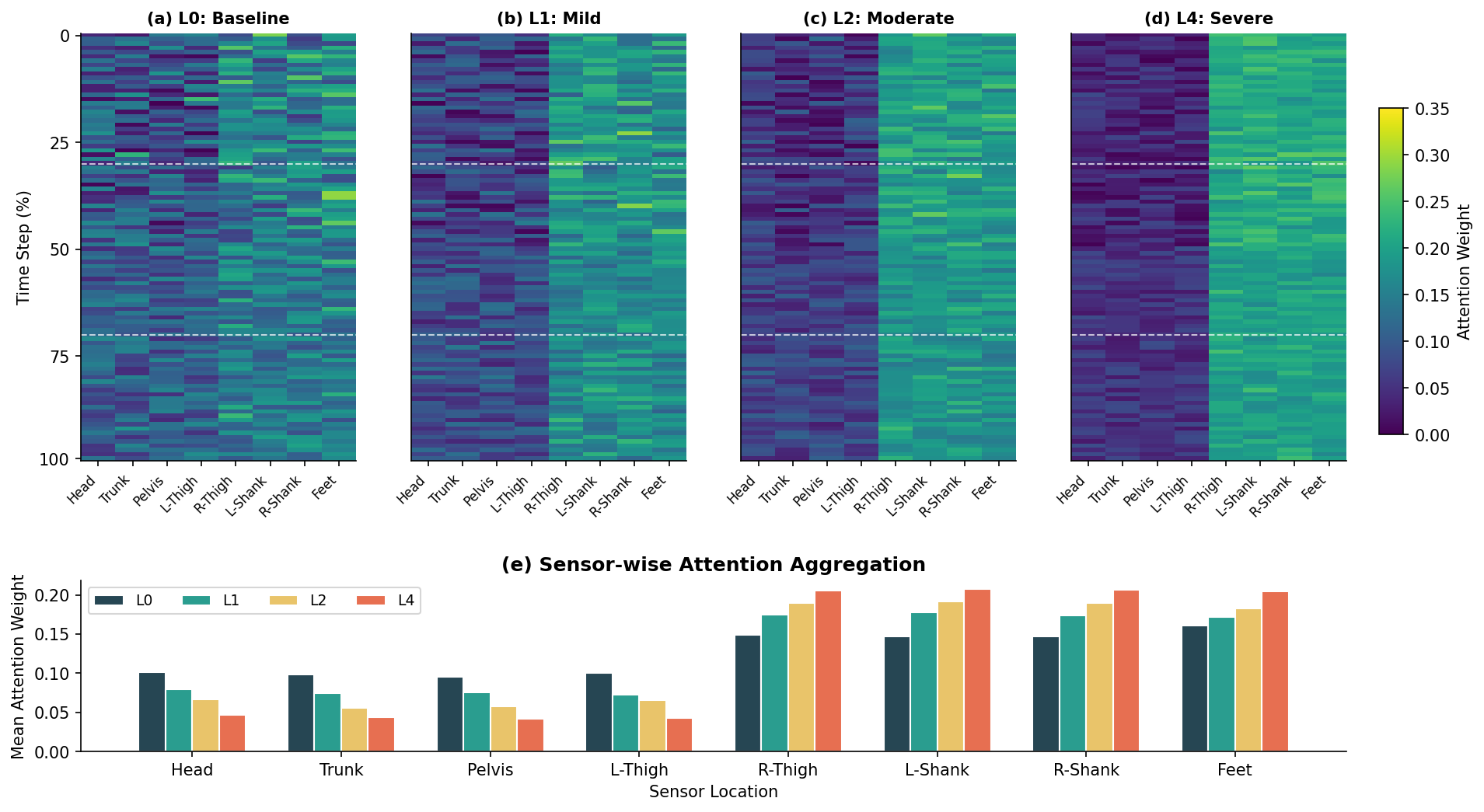

Supplement: Supplementary file 2 [file Supplementary_file_1.zip › Supplementary_Material/Figure6.jpg]

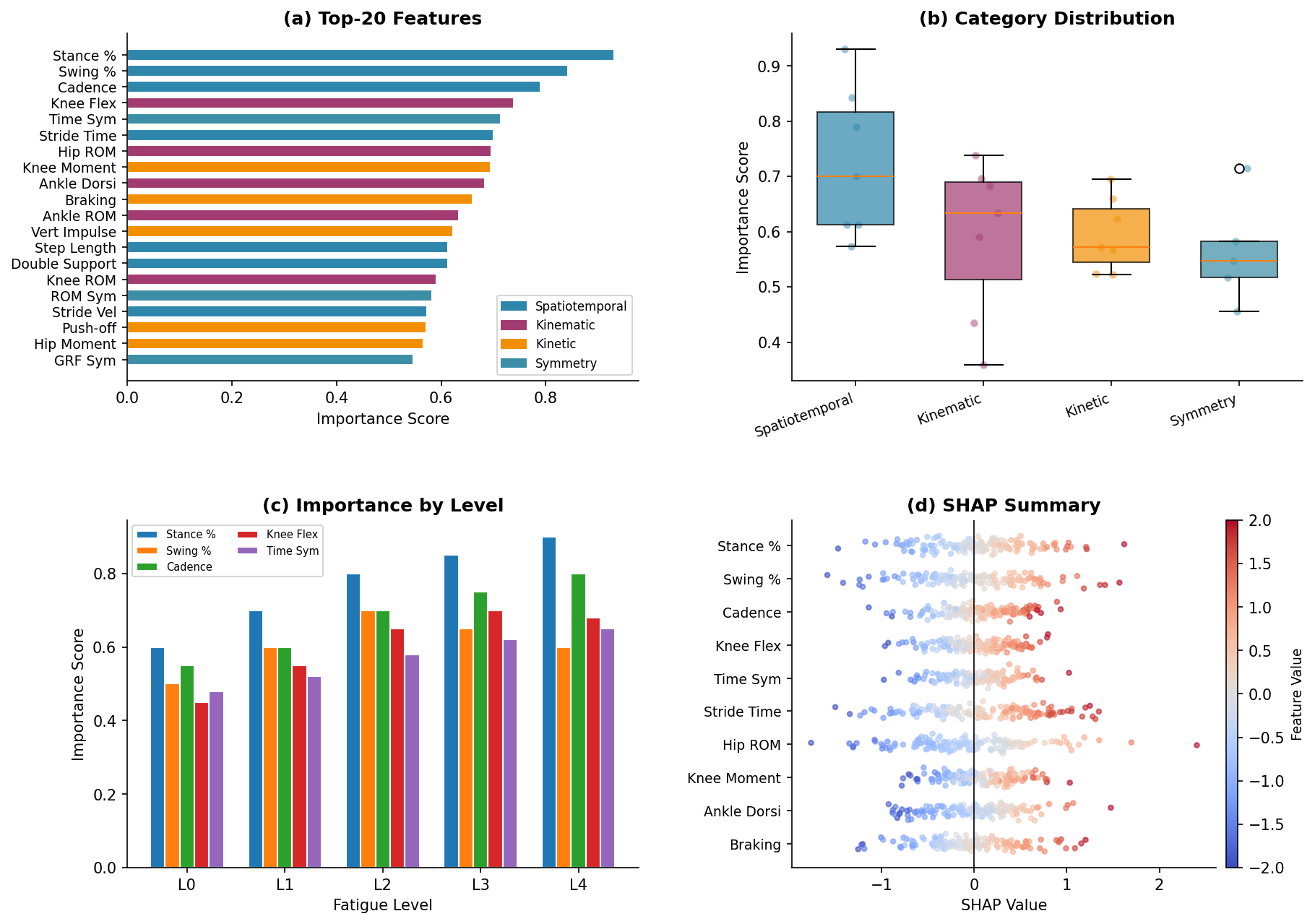

Supplement: Supplementary file 2 [file Supplementary_file_1.zip › Supplementary_Material/Figure7.jpg]
